# Low-Pressure Deuterium Storage on Palladium-Coated Titanium Nanofilms: A Versatile Model System for Tritium-Based Betavoltaic Battery Applications

**DOI:** 10.1021/acsami.3c06925

**Published:** 2023-08-17

**Authors:** Turkan Gamze Ulusoy Ghobadi, Yusuf Kocak, Ahsan Jalal, Yagmur Altinkaynak, Gulsah Celik, Tolga Semiz, Cihan Cakir, Bayram Butun, Ekmel Ozbay, Ferdi Karadas, Emrah Ozensoy

**Affiliations:** † Nanotechnology Research Center (NANOTAM), Bilkent University, Ankara 06800, Turkey; ‡ Department of Chemistry, Bilkent University, Ankara 06800, Turkey; § Department of Electrical and Electronics Engineering, Bilkent University, Ankara 06800, Turkey; ∥ Department of Physics, Bilkent University, Ankara 06800, Turkey; ⊥ National Nanotechnology Research Center (UNAM), Bilkent University, Ankara 06800, Turkey

**Keywords:** titanium hydride, deuterium, tritium, betavoltaic battery, hydrogen storage, catalysis, palladium

## Abstract

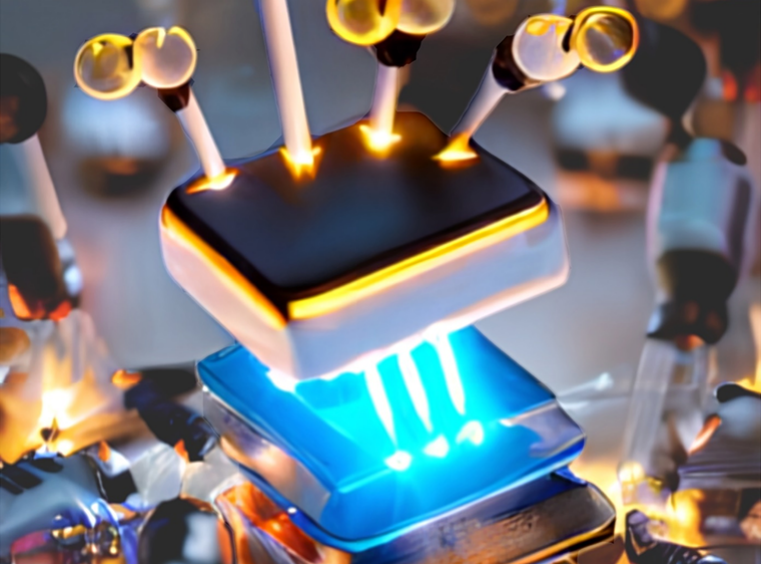

Deuterium (D_2_(g)) storage of Pd-coated Ti ultra-thin films at relatively low pressures is fine-tuned by systematically controlling the thicknesses of the catalytic Pd overlayer, underlying Ti ultra-thin film domain, D_2_(g) pressure (*P*_D2_), duration of D_2_(g) exposure, and the thin film temperature. Structural properties of the Ti/Pd nanofilms are investigated via XRD, XPS, AFM, SEM, and TPD to explore new structure-functionality relationships. Ti/Pd thin film systems are deuterated to obtain a D/Ti ratio of up to 1.53 forming crystallographically ordered titanium deuteride (TiD_*x*_) phases with strong Ti^*x*+^–D^*y*–^ electronic interactions and high thermal stability, where >90% of the stored D resides in the Ti component, thermally desorbing at >460 °C in the form of D_2_(g). Electronic interaction between Pd and D is weak, yielding metallic (Pd^0^) states where D storage occurs mostly on the Pd film surface (i.e., without forming ordered bulk PdD_*x*_ phases) leading to the thermal desorption of primarily DOH(g) and D_2_O(g) at <265 °C. D-storage typically increases with increasing Ti film thickness, *P*_D2_, *T*, and *t,* whereas D-storage is found to be sensitive to the thickness and the surface roughness of the catalytic Pd overlayer. Optimum Pd film thickness is determined to be 10 nm providing sufficient surface coverage for adequate wetting of the underlying Ti film while offering an appropriate number of surface defects (roughness) for D immobilization and a relatively short transport pathlength for efficient D diffusion from Pd to Ti. The currently used D-storage optimization strategy is also extended to a realistic tritium-based betavoltaic battery (BVB) device producing promising β*-*particle emission yields of 164 mCi/cm^2^, an open circuit potential (*V*_OC_) of 2.04 V, and a short circuit current (*I*_SC_) of 7.2 nA.

## 1 Introduction

Despite the fact that there exists widely accepted phase diagrams of hydrogen isotopes and bulk metals such as Ti and Pd,^1^ there is extremely limited knowledge on detailed hydrogen isotope uptake of nanometer scale multi-metallic ultrathin films at moderate/low pressures and temperatures. Furthermore, the chemical nature and relative thermal stabilities of the hydrogen isotopes stored in such nanometer scale systems, which are critical for the operational limitations of betavoltaic battery (BVB) devices,^2−9^ are also poorly known.

After the initial conceptual discovery of the BVB by Moseley and Fellow,^10^ associated technologies were first patented by Rappaport and Loferski at Radio Corporation of America in 1956.^11^ Tritium has a radioactive decay half-life of 12.32 years.^12^ Hence, unlike conventional electrochemical charge storage systems, tritium-based BVB can be utilized as long-lasting electron sources that can be operated within a wide temperature window and under challenging operational conditions^13^ with a prospect to substitute some of the existing low-power charge storage technologies exploited in pacemakers, underwater systems, communication, memory electronics, and aerospace applications.

Metal tritides prepared by storing tritium (_1_^3^T) in metals can be exploited as electron sources in BVB applications since _1_^3^T is a well-known β*-*particle (i.e., electron) emitter (_1_^3^T → _2_^3^He + *e*^–^ + *v*_e_^–^, where *v*_e_^–^ represents an electron antineutrino). Nanofabrication of _1_^3^T-based BVB devices requires the interfacing of the β-particle emitting material with the remaining multilayered semiconductor architecture, where the β-particle emitter can be in the form of a metal-tritide ultra-thin film.

Due to the radioactivity and the extremely high cost of _1_^3^T, deuterium (_1_^2^D) is commonly used as a proxy for _1_^3^T to investigate _1_^3^T storage properties of metals. This is an experimentally reasonable approach (which we also adopt in the current work) since former studies in the literature^14−16^ indicated that while the diffusion, permeability, and solubility coefficients of H isotopes in metals monotonically decrease with the increasing atomic mass of the relative isotopes, corresponding values for different isotopes are still rather comparable within a wide temperature and pressure range.

Thus, in the current work, we demonstrate that the deuterium uptake of Ti thin films decorated with Pd ultra-thin film overlayers can be systematically enhanced by carefully controlling Pd and Ti film thicknesses, D_2_ adsorption pressure, temperature, and duration. Furthermore, we examine the function of different metal sites (Pd vs Ti) in the ultra-thin film system as well as their chemical and electronic nature, and the relative thermal stabilities of the various forms of stored D species on/in the films which are critical parameters dictating the overall performance, longevity, and operational thermal limits of BVB devices. Finally, we illustrate that our findings on deuterium uptake could be successfully extended to tritium storage of Ti/Pd thin film systems, revealing promising β-emitting performances in realistic BVB devices.

## 2 Experimental Section

### 2.1 Materials

All of the chemicals were used as received without further purification. Additional details about materials are given in SI Section 1.1.

### 2.2 Ti/Pd Ultra-Thin Film Growth

In the current work, we use 4 different Pd film thicknesses, 2 different Ti film thicknesses, 3 different deposition temperatures, 6 different deposition durations, and 4 different deposition pressures, which span a parametric optimization space generated by a total number of 4 × 2 × 3 × 6 × 4 = 576 samples. Thus, by keeping in mind some of the critical requirements associated with the mass production of BV battery systems (i.e., low material cost, short manufacturing time, low manufacturing temperature, low *T*_2_(g) pressure), we carefully sampled relevant selected sections of this wide overall optimization space and justified our choices for these selections below.

Two different Ti film thickness (i.e., 300 and 500 nm) and four different Pd film thicknesses (i.e., 5, 10, 20, and 30 nm) were investigated in this study. In tritium-based BVB applications, β-particle emission flux of Ti-tritide films is predominantly governed by the tradeoff between the increasing attenuation of the β-particle emission with increasing Ti film thickness (due to the intrinsic absorption/scattering of the β-particles by the Ti matrix) and intensification of the β-particle emission flux with the increasing number of tritium atoms that can be accommodated in the thicker Ti films.

Accordingly, former studies in the literature^8^ suggest that Ti films with a thickness within 300–500 nm could be thin enough to allow a significant portion of the emitted β-particles to leave the Ti matrix, while they are thick enough to store a significant number of tritium atoms to enhance the β-particle flux.

Regarding the choice of the Pd film thicknesses, our preliminary experiments revealed that with the currently utilized e-beam evaporation protocols, Pd overlayers with thicknesses ≤5 nm did not completely wet/coat the Ti substrate (data not shown). On the other hand, a maximum Pd film thickness of 30 nm was also found to be appropriate in order to ensure effective D/T atom transport from the Pd film to the underlying Ti film by keeping the D/T atom diffusion path length at a reasonably small value and minimize the cost of the thin-film production. Additional details about materials are given in SI Section 1.2.

### 2.3 Deuterium Storage Experiments on Ti/Pd Thin Films

Technical details regarding the custom-design deuterium/tritium storage reactor are provided in SI Section 1.3 and Figure S1. Before the D_2_ storage experiments, Ti/Pd thin film samples were mounted into the reactor and the reactor was evacuated to <2 × 10^–8^ Torr at room temperature. During D_2_ uptake, D_2_(g) pressure in the reactor was adjusted to 2.5–20 Torr (i.e., pressures that ensure cost-effective mass production of *T*_2_(g)-based BV battery systems) and the temperature of the reactor was varied within 200–400 °C (i.e., low enough processing temperatures which are compatible with the nano-fabricated multilayer electron multiplier semi-conductor architectures to be interfaced with the β*-*particle emitter source in the BVB systems) for different D_2_ exposure durations of 15 min–10 h.

The absolute total amount of D_2_(g) stored by the thin films could be in principle estimated by comparing the change in deuterium pressure (Δ*P*_D2(g)_) at the beginning and end of the storage process. Since the absolute pressure transducer used in the deposition system had a sensitivity of 0.1 Torr, Δ*P*_D2(g)_ measurements could be only done for relatively large surface area samples (e.g., 2.0 cm × 2.0 cm). For the smaller surface area samples (0.7 ± 0.1 cm × 0.7 ± 0.1 cm), as described in detail below, the TPD technique was utilized which offered a much more accurate information regarding the *relative* amounts of deuterium stored in different films as well as various chemical forms in which deuterium was stored (e.g., D_2_, DH, DOH, D_2_O). On the other hand, the TPD technique did not provide information about the *absolute* amount of deuterium stored in the films. Thus, these two techniques were utilized in a complementary fashion in the current work.

### 2.4 Temperature Programmed Desorption

TPD experiments were conducted in a custom-design spectroscopic reactor (whose details were described elsewhere)^17^ equipped with a Stanford Research Systems, RGA 200 quadrupole mass spectrometer (QMS). In the TPD experiments, the reactor containing the mounted sample was initially evacuated to a pressure of ca. 1 × 10^–6^ mbar at room temperature. Then, the samples were heated from room temperature to 700 °C with a linear ramp rate of 12 °C/min in vacuum. Relevant desorption channels corresponding to deuterium (D) containing species such as *m*/*z* = 3 (DH), *m*/*z* = 4 (D_2_), *m*/*z* = 18 (DO/H_2_O), *m*/*z* = 19 (DOH), *m*/*z* = 20 (D_2_O) were simultaneously recorded as a function of desorption time/temperature during the TPD experiments. Relative amounts of deuterium atoms originating from these different deuterium containing species were calculated by comparing integrated desorption signals (i.e., areas) below the TPD traces of the corresponding desorption channels which are further normalized by the corresponding surface areas of the thin films. Integrated desorption signals for *m*/*z* = 4 (D_2_) and *m*/*z* = 20 (D_2_O) were multiplied by 2 as these desorption species contained two deuterium atoms per desorbed molecule. Relatively minor contributions from other mass spectroscopic fragmentation channels were neglected. It should be noted that the sample holder used in the TPD system allowed a maximum thin film size of 0.8 cm × 0.8 cm. Thus, TPD analysis experiments were carried out using films with typical dimensions of 0.7 ± 0.1 cm × 0.7 ± 0.1 cm.

### 2.5 Material Characterization

Surface morphology, elemental composition, electronic structure, and the crystal structure of the deposited Ti/Pd ultra-thin film samples before and after deuterium storage were investigated via scanning electron microscopy (SEM), X-ray photoelectron spectroscopy (XPS), XPS depth profiling, valence-band XPS (VB-XPS), grazing-incidence X-ray diffraction (GIXRD), and atomic force microscopy (AFM). Experimental parameters regarding these measurements are provided in SI Section 1.4.

## 3 Results and Discussion

### 3.1 Surface Morphology of the Ti/Pd Thin Films

**Figure 1:**
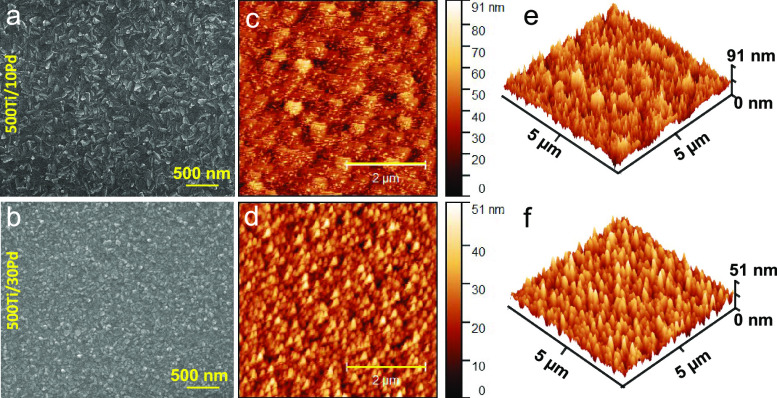
(a,b) SEM and (c–f) AFM images of pristine 500Ti/10Pd (top row) and 500Ti/30Pd (bottom row) thin film samples. AFM scale bar: 2 μm.

Figure 1a–f shows SEM and AFM images of 500Ti/10Pd and 500Ti/30Pd films corresponding to some of the best-performing films in the current work, revealing their surface morphology and roughness as a function of Pd film thickness. SEM images (Figure 1a,b) indicate the presence of flakelike 2D-platelet Pd nanostructures on both surfaces. The roughness measurements were carried out via AFM (Figure 1c–f) by utilizing a sampling area of ca. 0.005 mm which also revealed decreasing surface roughness with increasing Pd film thickness where mean surface roughness (*R*_a_) decreased from 8.9 to 5.3 nm with increasing Pd film thickness.

### 3.2 Elemental Composition and Electronic Structure of the Ti/Pd Thin Films

**Figure 2:**
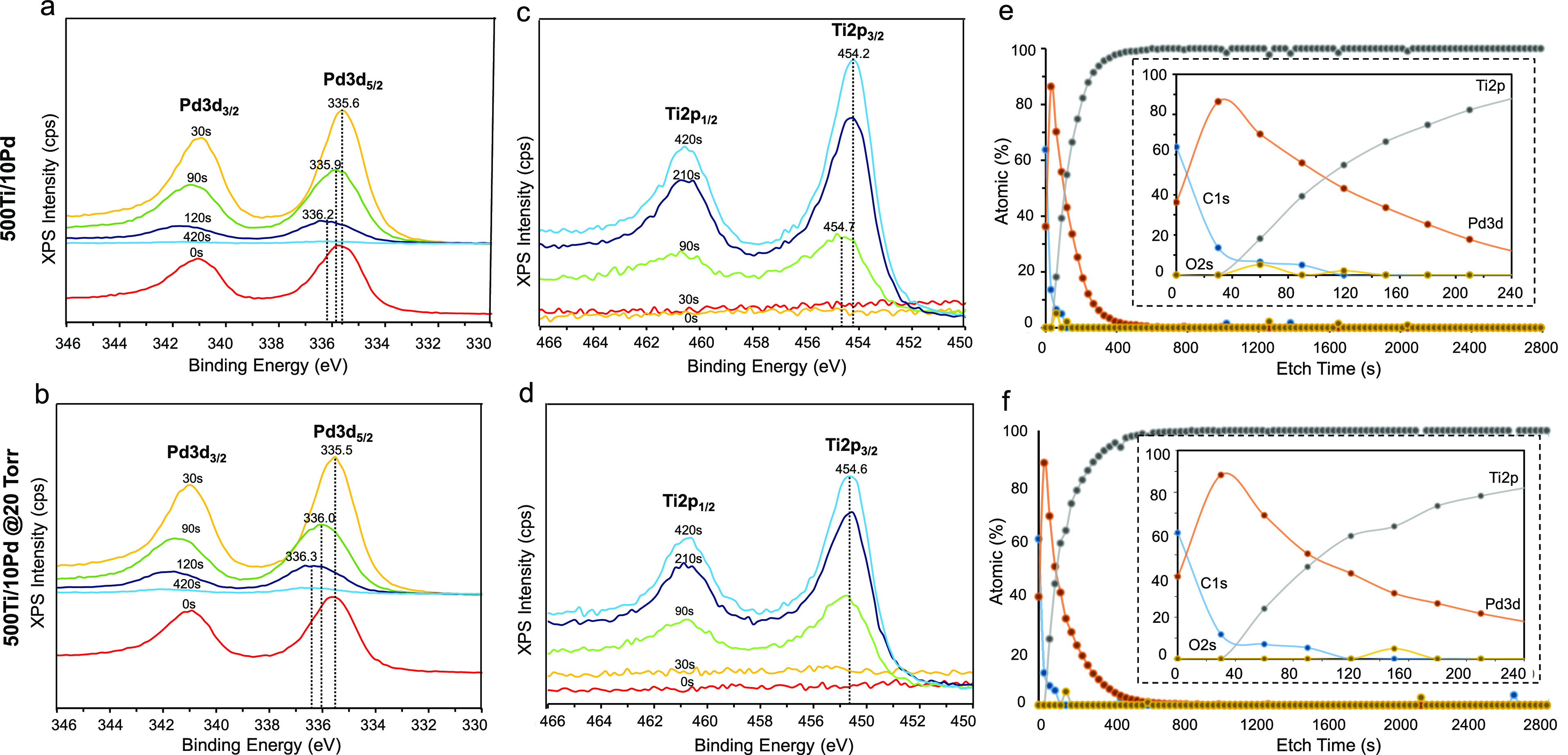
Pd3d (a,b), Ti2p (c,d), and depth profiling XPS data as a function of sputtering time (0–420 s) (e,f), for pristine and D_2_-exposed (20 Torr, 300 °C, 15 min) 500Ti/10Pd thin films, respectively.

**Figure 3:**
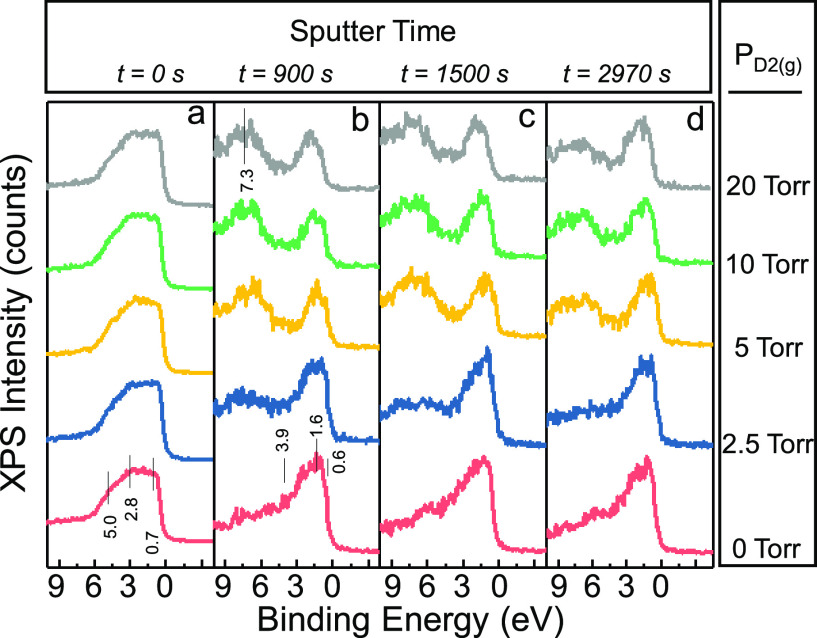
VB-XPS data for 500Ti/10Pd as a function of Ar^+^(g) sputter time: (a) 0 s, (b) 900 s, (c) 1500 s, (d) 2970 s. D_2_(g) exposure was performed at *P*_D2(g)_ = 0, 2.5, 5, 10, 20 Torr, 300 °C, 15 min.

Detailed elemental composition and the oxidation states of Pd and Ti species in the 500Ti/10Pd thin films before and after D_2_(g) storage at 20 Torr, 300 °C, and 15 min were investigated via XPS-depth profiling (Figures 2a–f, and S2) and VB-XPS (Figure 3a–d) techniques.

Figure 2a illustrates that before the sputtering process (i.e., *t* = 0 s), the Pd3d_5/2_ feature is located at 335.6 eV (with a spin–orbit splitting of 5.3 eV) which is consistent with the presence of metallic (Pd^0^) palladium species.^18^ On the other hand, with the increasing sputtering time within 30–420 s, the Pd3d_5/2_ signal of the pristine 500Ti/10Pd surface shifts to a higher B.E. value of 336.2 eV (i.e., +0.6 eV shift). Considering the increase in the oxygen atom content at the Pd–Ti interface evident by the corresponding XPS depth profiling data (see O2s data for *t* = 30–150 s in the inset of Figure 2e), it is apparent that metallic Pd species on the very top surface of the 500Ti/10Pd thin film gradually transform (at least partially) to Pd^*x*+^ species^19,20^ at the Pd–Ti interface, due to the presence of a thin TiO_*x*_ overlayer which is formed during the Ti film deposition process as a result of the reaction between oxophilic Ti metal and the oxygen-containing background gases. It should be noted that due to the low X-ray photoemission cross-section of the O2s species, while the influence of the TiO_*x*_ overlayer at the Pd–Ti interface is visible in the Pd3d spectra within sputtering times of *t* = 90–420 s (Figures 2a and S2a), O2s species are only detected within *t* = 60–150 s of the corresponding XPS depth profiling data (Figure 2e).

As an alternating explanation, the +0.6 eV shift in the Pd3d_5/2_ feature of the pristine 500Ti/10Pd surface (Figure 2a) with decreasing Pd film thickness can be also attributed to the surface core level shift (SCLS) phenomena^21,22^ in the Pd thin film overlayer. It was demonstrated by Rodriquez and Goodman that Pd3d_5/2_ feature of the Pd ultrathin films grown on single crystal metals such as Mo(110), W(110), Re(0001) show a +0.5 eV shift as the Pd surface coverage decreases from ≥8 ML (i.e., a Pd film thickness ca. 2.1 nm) to 0 ML. Such SCLS phenomena observed for Pd ultrathin films grown on metallic substrates were attributed to the increase in the coordination number of the Pd adatoms with increasing Pd film thickness, in addition to the strong electronic interactions between the metallic Pd (i.e., Pd^0^) ultrathin film overlayers and the underlying metallic substrates originating from the increasing transfer of electrons from the occupied orbitals of the metallic Pd overlayers into the empty electronic states of the metallic substrate at small Pd film thicknesses.

Figures 2c and S2c reveal that corresponding changes in the Ti2p XPS data for the pristine 500Ti/10Pd thin films are also consistent with both of the arguments mentioned above. Accordingly, with the increasing sputtering time (i.e., decreasing Pd film thickness), the metallic Ti2p_3/2_ (Ti^0^) feature at 454.7 eV gradually shifts to a lower B.E. of 454.2 eV, revealing a −0.5 eV shift in line with increasing electron transfer from Pd to Ti. Alternatively, with increasing sputtering time, TiO_*x*_ interfacial layer is gradually depleted, leading to the attenuation of Ti^*x*+^ states and the observed redshift in Ti2p states.

Exposure of D_2_(g) to the 500Ti/10Pd thin film system did not result in significant differences in the trends observed in the corresponding Pd3d XP spectra (Figures 2b and S2b) nor in the elemental composition data obtained from XPS-depth profiling analysis (Figure 2f) as compared to that of the pristine 500Ti/10Pd sample (Figures 2c,f and S2b). Currently used D_2_(g) exposure temperature of 300 °C (573 K) is above the critical temperature of 566 K^23^ leading to the formation of a PdD_*x*_ system likely composed of weakly interacting D atoms dissolved in the Pd lattice rather than a well-defined metal hydride phase composed of strong Pd–D interactions. Thus, the introduction of D_2_(g) did not lead to a drastic influence on Pd3d XPS features (Figure 2b) as compared to that of the pristine 500Ti/10Pd (Figure 2a). On the other hand, a comparison of the Ti2p spectra for the 500Ti/10Pd surface with (Figures 2d and S2d) or without (Figures 2c and S2c) D_2_(g) exposure show discernible differences. Namely, D_2_(g) exposed 500Ti/10Pd yields a constant but a relatively blue-shifted Ti2p signal at 454.6 eV within sputtering times of *t* = 0–420 s, which is consistent with a strong interaction between Ti and D species in the titanium deuteride (TiD_*x*_) domains, giving rise to electron donation from Ti to D species and formation of Ti^*x*+^ states at all of the measured film thickness in Figures 2d and S2d. Here, a minor contribution from TiO_*x*_ interfacial species cannot be ruled out either.

Former studies reported that the valence band structure of Pd ultrathin films grown on various metallic single crystal substrates (e.g., Ta(110), Nb(110), W(110), Cu(111), Ag(111), Au(111), Al(111))^24^ reveal significant differences with respect to bulk Pd. However, these changes are rather insensitive to the choice of the metallic substrate. For a 1 ML Pd film on Au(111), a major B.E. peak at 1.6 eV below the Fermi level was observed with a weak shoulder at 0.4 eV. Increasing the Pd film thickness on Au(111) to 5 ML resulted in a broad Pd-related B.E. signal at 0–5 eV. These features are consistent with the current VB-XPS data for *t* = 0 s in Figure 3a which correspond to the 500Ti/10Pd surface before sputtering. Since the typical surface sensitivity of the XPS technique is ≤10 nm, *t* = 0 s spectra in Figure 3a are predominantly due to the Pd overlayer with a 10 nm thickness and the contribution from the underlying Ti film is minor. Furthermore, VB-XPS data for *t* = 0 s obtained after D_2_(g) exposures at 2.5–20 Torr did not lead to any significant changes in the VB-XPS as compared to that of the pristine 500Ti/10Pd surface (i.e., *P*_D2(g)_ = 0 Torr). This observation is in very good agreement with the Pd3d XPS results discussed above (Figure 2), suggesting a weak interaction between Pd and D and/or a limited D-storage in the Pd overlayer of the 500Ti/10Pd thin film.

VB-XPS data in Figure 3b–d for sputtering times *t* ≥ 900 s correspond to complete removal of the Pd overlayer and represent the electronic structure of the underlying Ti film as a function of depth and deuterium pressure. VB-XPS results for *t* ≥ 900 s of the pristine 500Ti/10Pd surface reveal a weak shoulder at 0.6 eV, a strong peak at 1.6 eV, and an asymmetric tail extending between 3.9–8.0 eV. Former ultraviolet photoelectron spectroscopy (UPS) studies on Ti(0001)^25^ and Ti films^26^ as well as electron energy loss spectroscopy (EELS) studies in the literature are in line with these features suggesting the presence of mostly metallic Ti^0^ species with a minor contribution from background hydrogen adsorbed on/near the surface region of Ti during Ti film growth. On the other hand, upon exposure of D_2_(g), VB-XPS data for *t* ≥ 900 s in Figure 3b–d depict the evolution of an additional intense feature at ca. 7 eV, indicating the storage of D in the Ti layer and formation of a TiD_*x*_ hydride phase, revealing a strong interaction between Ti and D, which is in very good accordance with the stark spectral changes in the corresponding Ti2p XPS data observed upon D_2_(g) exposure (Figure 3).

### 3.3 Crystal Structure of the Ti/Pd Thin Films

**Figure 4:**
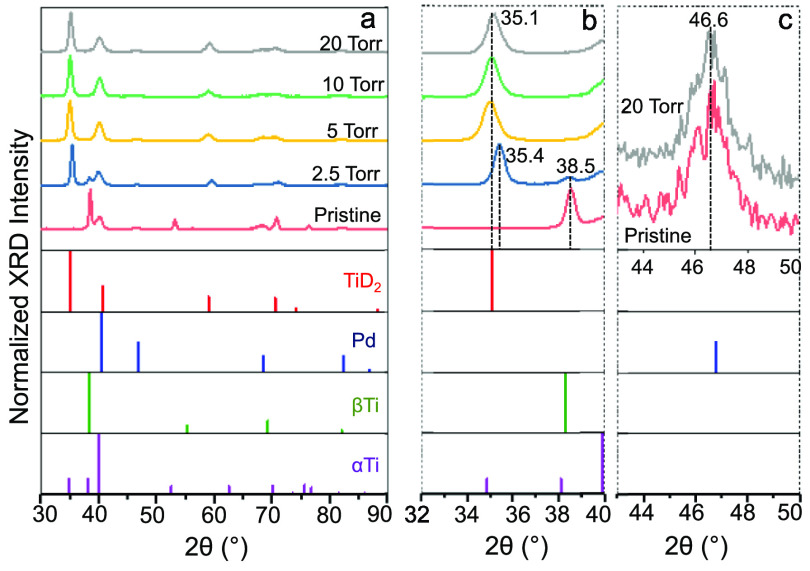
XRD data for 500Ti/10Pd as a function of D_2_(g) exposure performed at *P*_D2(g)_ = 0, 2.5, 5, 10, 20 Torr, 300 °C, 15 min. (a) Full diffractograms, (b) detailed view of primary Ti and TiD_*x*_ signals, and (c) detailed view of a selected Pd signal.

Figure 4a–c shows the XRD data for the pristine and D_2_(g)-exposed 500Ti/10Pd thin films. Pristine 500Ti/10Pd thin film is characterized by metallic Pd (JCPDS Card No. 00-001-1201) and metallic (bcc) β-Ti phases^27^ (JCPDS Card No. 01-074-7075), where the metallic α-Ti phase^27^ (JCPDS Card No: 98-005-3784) is mostly absent. Upon exposure to D_2_(g) at 2.5 Torr, a new diffraction signal appears at 2θ = 35.4° (Figure 4b) which converges to 35.1° for *P*_D2(g)_ ≥ 5 Torr, revealing gradual expansion of the metallic unit cell due to the formation of crystalline titanium deuteride domains (JCPDS card no. 04-014-5373). This finding is in good harmony with the current XPS (Figure 2) and VB-XPS results (Figure 3), indicating a strong interaction between Ti and D upon exposure of 500Ti/10Pd thin films to D_2_(g).

In contrast, Figure 4c depicts that exposure to D_2_(g), even at the highest pressure utilized here (i.e., 20 Torr), does not alter the Pd diffraction signals (e.g., see 46.6° diffraction signal of Pd which is not overlapping with any Ti or TiD_*x*_ signals), suggesting a weak interaction between Pd and D as well as a lack of ordered Pd-deuteride phases nor significant D-storage in the Pd overlayer. Lack of D in the Pd overlayer may originate from the weaker Pd-D interactions at the Pd surface as opposed to that of the bulk of Pd. As a result, relatively weakly bound and mobile D atoms dissolved in the Pd bulk matrix can readily be transported to the underlying Ti film, where they can strongly interact with Ti and form ordered TiD_*x*_ domains.

### 3.4 Optimization of D_2_(g) Storage of Ti/Pd Thin Films via TPD

Bulk Ti metal typically exists in the form of one of the two different allotropes (or in the form of their mixtures) which are the low-temperature hexagonal close-packed (hcp) α phase, or the high-temperature body-centered cubic (bcc) β phase.^27^ Upon introduction of hydrogen on Ti, once hydrogen dissociatively adsorbs and the generated H atoms permeate through the surface native oxide layer; they can either diffuse and redistribute themselves toward the bulk or be trapped by the Ti matrix. Note that the diffusion coefficient of H in Ti-hydrides at room temperature^28^ (ca., 4 × 10^–12^ cm^2^ s^–1^) is about two orders of magnitude lower than that of the metallic Ti matrix. Furthermore, H transport in the Ti matrix is strongly affected by irreversible trapping phenomena originating from lattice imperfections and crystal defects such as dislocations, precipitates, and grain boundaries.^29^ These irreversible trapping sites facilitate the nucleation and growth of Ti-hydride phases.

Former studies on dehydrogenation of titanium hydrides (TiH_*x*_)^29−32^ reported that for *x* = 2, a face-centered tetragonal (FCT) ε-hydride (c/a < 1) was observed which transformed into the most stable face-centered cubic (FCC) δ-hydride phase within 1.5 < *x* < 1.99. Decrease in hydrogen content (1.0 < *x* < 1.5) led to the formation of a mixture of (FCC) δ-hydride and an FCT β-hydride (c/a >1) phases. Further decline in the hydrogen content (i.e., 0.2 < *x* < 1.0) resulted in the existence of FCT β-hydride as a single phase. On the other hand, extremely low hydrogen concentrations (i.e., 0 < *x* < 0.2) revealed the co-existence of FCT β-hydride, α-hydride, and metallic α-Ti phases, where FCT β-hydride and α-hydride phases subsequently vanished upon continuous dehydrogenation within this concentration interval to form the ultimate metallic α-Ti after complete dehydrogenation.

The hydrogen isotope (i.e., H, D, T) storage process in Ti films often requires relatively high temperatures and pressures. This is partly due to the fact that Ti metal is not very efficient in low-temperature activation of molecular hydrogen and transport of H atoms into the bulk.^33,34^ For example, in order to achieve a saturation D/Ti atomic ratio of ca. 1.9, Ti films need to be exposed to D_2_(g) pressures as high as 0.1–10 bar at elevated sample temperatures (e.g., 400 °C).^35−37^ Before the hydrogen isotope storage process, Ti film surfaces are typically annealed at high temperatures (e.g., >800 °C) in vacuum in an attempt to remove surface contaminations. Furthermore, the hydrogen isotope storage process on Ti thin films also typically requires elevated temperatures due to the need for the efficient activation of the H–H/D–D/T–T bonds via dissociative adsorption as well as diffusion of the generated adsorbed atomic hydrogen isotopes toward the bulk of Ti and formation of the metal hydride/deuteride/tritide phases.^38,39^ This is also linked to the fact that the solubilities of hydrogen isotopes in Ti also increase with increasing temperature. However, such high-temperature pretreatment and hydrogen isotope storage protocols can also trigger the formation of surface and/or bulk Ti oxides as a result of the reaction between the oxophilic Ti metal and oxygen-containing background gases such as O_2_, H_2_O, etc.^40^ Presence of surface/bulk titanium oxide (TiO_*x*_) phases can increase the activation energy required for the diffusion and transport of hydrogen^29^ isotopes toward the bulk and suppress the ultimate hydrogen isotope storage capacity of the Ti-containing phases. For example, diffusion coefficients of hydrogen in metallic Ti polymorphs were reported to be 10^5^–10^15^ times greater than that in titania (i.e., TiO_2_/TiO_*x*_) polymorphs.^29^ Thus, it is essential to minimize the exposure of pristine Ti films to air or other reactive gases. Moreover, metal hydride phases may also unfavorably deteriorate the mechanical integrity of the thin film by triggering crack formation and crack propagation^29,41,42^ (i.e., hydrogen embrittlement and hydride-induced cracking).

In light of the abovementioned requirements, an ideal hydrogen isotope storage thin-film system should facilitate dissociative adsorption of molecular hydrogen and transport of H/D/T atoms to the bulk by assisting their storage in the form of MH_*x*_/MD_*x*_/MT_*x*_ phases at relatively low temperatures and pressures, while offering passivation of the surface against chemical contaminations and mechanical integrity loss. The Pd ultra-thin film overlayer on a Ti thin film serves as an excellent candidate to satisfy these requirements since the Pd overlayer is a multifunctional component that can (i) prevent oxidation of the underlying Ti matrix by forming a relatively inert passivation layer, (ii) catalyze H_2_(g)/D_2_(g)/T_2_(g) dissociative adsorption at low temperatures, low pressures, and short times,^43^ (iii) facilitate the H/D/T diffusion toward the Ti matrix at relatively low temperatures,^44^ and (iv) enhance long-term mechanical durability by suppressing hydrogen embrittlement and hydride-induced cracking.

PdH_*x*_ phase diagram at H_2_ pressure of 1 mbar at 298 K typically involves α-Pd hydride phase at low H concentrations (i.e., *x* < 0.017) and α’-Pd hydride phase at high H concentrations (i.e., *x* > 0.60), or a mixture of α and α′ Pd hydride phases at intermediate H concentrations, where H is located at the interstitial octahedral holes of the fcc Pd matrix in all cases.^23^ Above the critical temperature of 566 K (i.e., at the temperatures investigated in the current work), distinction between α and α′ phases vanish, and the PdH_*x*_ system can be described via a lattice gas model.^23^

Ultra-high vacuum (UHV) studies on hydrogen adsorption on Pd single crystal or Pd ultra-thin film surfaces reported the dissociative adsorption of molecular hydrogen leading to strongly bound chemisorbed hydrogen (desorbing at 250–400 K) as well as relatively weaker bound bulk/subsurface absorbed H species (desorbing at *T* < 250 K with zeroth-order desorption kinetics).^43−49^ It was reported that hydrogen uptake of Pd(111) increased by a factor of 5 with increasing the adsorption temperature from 90 to 300 K at a hydrogen pressure of 2.8 × 10^–6^ Torr.^43^ A similar behavior was also observed for hydrogen uptake of Pd thin films grown on the metallic single crystal Ta(100) substrate under UHV conditions.^43^ In addition, the generation of defects on the Pd(111) surface via Ar^+^ sputtering at 115 K further increased the population of bulk/subsurface H species by a factor of 4.^49^ However this latter effect is not seen for sputtered Pd(111) surfaces which were annealed and healed at 298 K prior to hydrogen adsorption.^43^

It was also reported that hydrogen adsorption on Pd(111) at *T* ≥ 261 K under UHV conditions also led to a high-temperature hydrogen desorption tail located at ca. 800 K, suggesting activated storage of H in the bulk of Pd.^43^ It should be noted that activation barriers for hydrogen desorption were also shown to depend on the hydrogen concentration in the surface, subsurface, and bulk regions of Pd.^44^

Hydrogen adsorption studies on Pd films deposited on Nb and Ta foils under UHV conditions indicated that hydrogen uptake increased as the Pd film thickness increased from 1 monolayer (ML) (forming an incommensurate fcc(111) Pd overlayer) to 5 ML (i.e., corresponding to a Pd thickness of ca. 1.3 nm) and this maximum hydrogen uptake for 5 ML Pd did not increase further with increasing Pd film thickness to 100 ML (i.e., corresponding to a Pd thickness of ca. 26 nm).^50^

**Figure 5:**
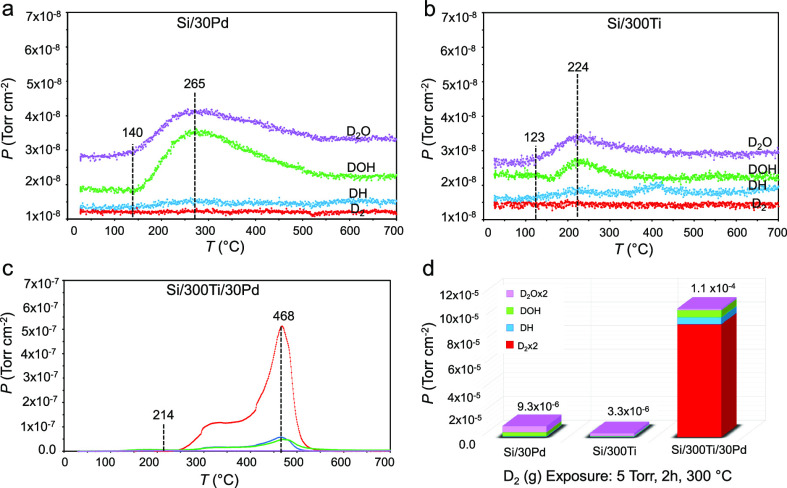
Surface area normalized TPD data for deuterated (a) Si/30Pd, (b) Si/300Ti, (c) Si/300Ti/30Pd thin films, (d) corresponding surface area normalized integrated TPD desorption signals of D-containing desorption species. D_2_(g) storage experiments were carried out at 5 Torr and 300 °C for 2 h for all samples.

Control experiments presented in Figure 5 show TPD data obtained after deuteration (under the same conditions, *P*_D2(g)_ = 5 Torr, 300 °C, and 2 h) of a 30 nm thick Pd film deposited on Si(100) (Si/30Pd, Figure 5a), a 300 nm thick Ti film deposited on Si(100) (Si/300Ti, Figure 5b), and a 30 nm thick Pd overlayer film deposited on a 300 nm thick Ti thin film grown on Si(100) (Si/300Ti/30Pd, Figure 5c).

These results clearly indicate that the presence of both Pd and Ti components are essential for effective deuterium storage, where lack of the Ti storage domain results in an attenuation of total D storage by a factor of 12 (Figure 5a,d) and absence of the catalytic Pd overlayer that is responsible for (i) effective D_2_ adsorption, (ii) D–D bond cleavage, and (iii) surface passivation leads to a decrease in the total D storage by a factor of 32 (Figure 5b,d) as compared to that of the Si/300Ti/30Pd thin film system (Figure 5c,d).

Synergistically operating Pd overlayers on Ti films of the Si/300Ti/30Pd system not only enhances total deuterium uptake but also stabilizes the stored D species via strong Ti–D interactions in the Ti domains evident by the significantly higher primary desorption maximum (468 °C) of Si/300Ti/30Pd (Figure 5c) as compared to that of Si/30Pd and Si/300Ti samples (<300 °C, Figure 5a,b). In addition, initiation of the desorption of D-containing species occurs at a higher temperature of 214 °C on Si/300Ti/30Pd whereas it takes place at ≤140 °C on Si/30Pd and Si/300Ti surfaces. This latter point is particularly important for the operational safety and versatility of BV battery systems as higher deuterium/tritium desorption temperatures enable utilization of BV systems within a broader thermal window enabling their deployment under more challenging operational conditions.

Moreover, in light of the current XPS, VB-XPS, and XRD characterization results presented in Figures 2–4, as well as the relative total D-storage data given in Figure 5d, it can be argued that almost all (≥92%) of the D storage in the Si/300Ti/30Pd system occurs in the Ti component. It is also apparent that in Si/30Pd and Si/300Ti systems, deuterium storage is limited to mostly D adsorption on the film surface rather than D storage in the bulk. This is due to the fact that these systems do not reveal any significant D_2_(g) desorption but rather release D_2_O(g) and DOH(g) (Figure 5a,b) as a result of D–H exchange between adsorbed D atoms on the surface and the H_2_O molecules adsorbed on these surfaces upon exposure of these samples to air prior to the TPD experiments.

**Figure 6:**
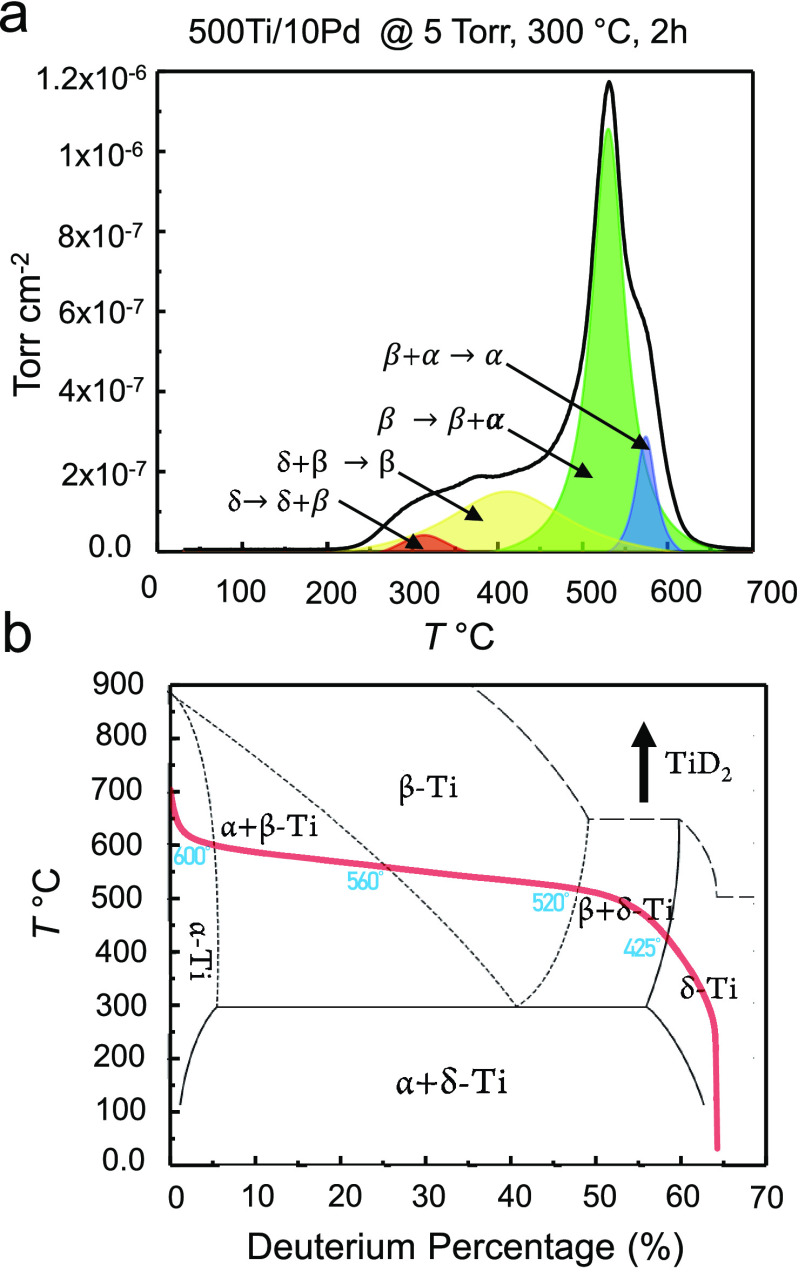
(a) Deconvolution of the D_2_ (g) (*m*/*z* = 4) TPD signal of 500Ti/10Pd sample which was initially deuterated at *P*_D2(g)_ = 5 Torr, 300 °C, and 2 h. (b) Titanium-deuterium phase diagram adapted from ref (34). Red curve is a guide to the eye for the observed thermally induced phase transitions of TiD_1.38_ occurring due to the deuterium desorption from 500Ti/10Pd thin film system given in part (a).

Detailed analysis of TPD profiles obtained after deuteration of Ti/Pd thin films (Figure 6) offer valuable information about the thermally induced polymorphic phase changes occurring in the titanium deuteride domains. Figure 6a depicts the deconvolution of the TPD desorption peaks (using a different Gaussian peak for each phase transition process) obtained for the 500Ti/10Pd sample, i.e., one of the best performing samples studied in the current set of TPD experiments, which was deuterated at *P*_D2(g)_ = 5 Torr, 300 °C, and 2 h. As will be illustrated below, this particular sample had a D/Ti of 1.38 corresponding to a percent D storage of 58% D in its stoichiometry. In light of the titanium–deuterium phase diagram presented in Figure 6b which is adapted from a former work in the literature,^34^ it can be argued that the first desorption peak at 314 °C in Figure 6a is associated with a phase transition from the δ-titanium deuteride phase to a mixture of δ *+* β-titanium deuteride phases. Accordingly, in Figure 6a, the desorption peak at 425 °C can be assigned to a phase transition from δ *+* β-titanium deuteride to β-titanium deuteride, while the most intense desorption maximum at 520 °C can be ascribed to a phase transition from β-titanium deuteride to α *+* β-titanium deuteride. Finally, the remaining desorption feature at 560 °C can be appointed to the transformation of the α + β-titanium deuteride to an α-titanium deuteride phase.

**Figure 7:**
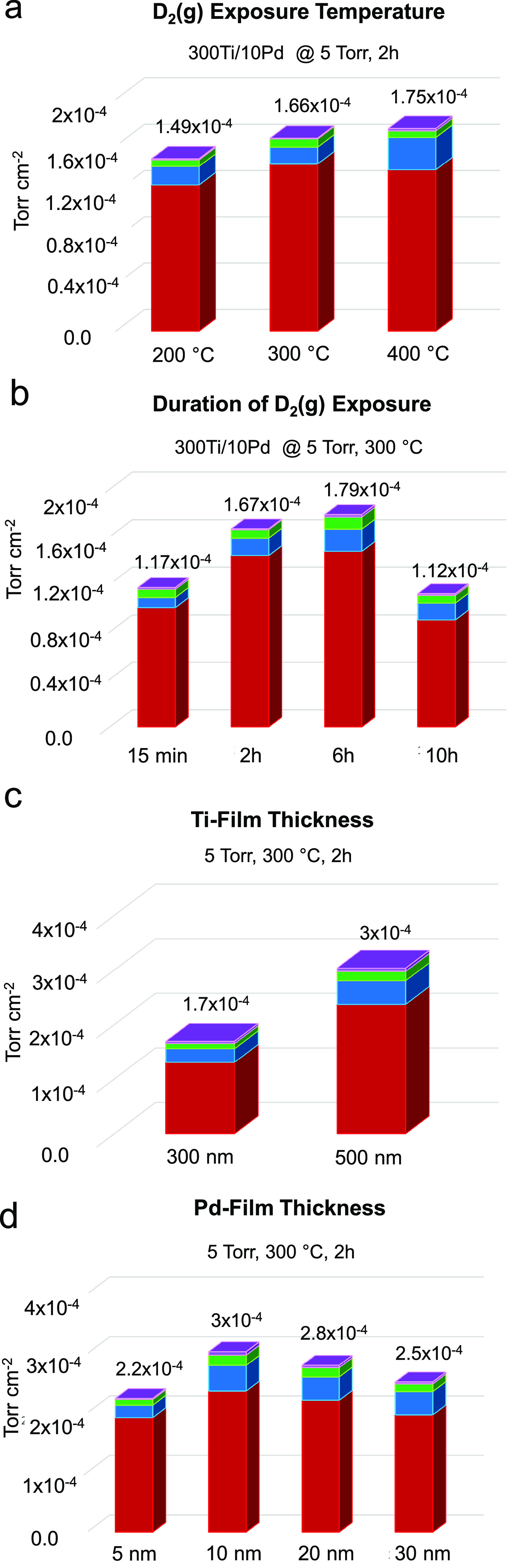
Surface area normalized integrated TPD desorption signals of D-containing desorption species in different D_2_(g) storage optimization runs. Influence of (a) storage temperature, (b) duration of D_2_(g) exposure, (c) Ti film thickness, (d) Pd film thickness. Color Bars; Red: D_2_ × 2, Blue: DH, Green: DOH, Purple: D_2_O × 2.

Figure 7 summarizes some of the main findings of the deuterium storage optimization experiments carried out using selected sets of optimization parameters. Corresponding TPD profiles for these experiments are also provided in Figure S3. First, influence of D_2_(g) exposure temperature were investigated within 200–400 °C on 300Ti/10Pd films using *P*_D2(g)_ = 5 Torr and D_2_(g) exposure duration of 2 h (Figure 7a). It is apparent that total D storage increases only slightly with increasing temperature. Accordingly, 300 °C was used in the forthcoming storage experiments in an attempt to provide sufficiently high temperature for D_2_ activation and storage which is also a relatively low temperature that is preferable for mass production of BV battery systems.

Second, we studied the effect of D_2_(g) exposure duration within 0.25–10 h on 300Ti/10Pd films using *P*_D2(g)_ = 5 Torr at a temperature of 300 °C (Figure 7b). This set of experiments revealed that D storage increased as the D_2_(g) exposure duration increased from 0.25 to 2 h and stayed at this value for 6 h. However, upon increasing D_2_(g) exposure duration to 10 h, D storage showed a visible decline. In other words, upon extensive durations (10 h) of D_2_(g) exposure at 5 Torr and 300 °C, desorption of D-containing species started to prevail (possibly due to a slowly occurring phase transition in the Ti-hydride domains) resulting in a decrease in the total D uptake. Based on the results presented in Figure 7b, a D_2_(g) exposure duration within 2–6 h could be chosen in the forthcoming optimization steps. Thus, for the sake of minimizing the process time, we proceeded with a D_2_(g) exposure duration of 2 h in the next set of optimization experiments.

Figure 7c emphasizes the impact of the Ti film thickness (300 nm vs 500 nm) on D-storage of Ti/Pd films with a Pd overlayer thickness of 10 nm, studied at *P*_D2(g)_ = 5 Torr and D_2_(g) exposure duration of 2 h at 300 °C. As expected, increasing Ti film thickness increased the total D uptake almost in a linear fashion. Hence, in the forthcoming optimization tests, a Ti film thickness of 500 nm was exploited.

Next, we examined the variation in the total D storage as a function of the thickness of the catalytic Pd overlayer (Figure 7d) within 5–30 nm for Ti/Pd with a Ti overlayer thickness of 500 nm, studied at *P*_D2(g)_ = 5 Torr and D_2_(g) exposure duration of 2 h at 300 °C. Figure 7d shows that the highest D-storage was obtained for a Pd film thickness of 10 nm while either smaller or greater Pd film thicknesses than 10 nm led to the attenuation of the D storage. It is apparent that a small Pd film thickness of 5 nm does not fully wet the underlying Ti surface due to the formation of small Pd clusters during the Pd overlayer deposition process resulting in the presence of only a limited number of catalytically active Pd sites on the Ti surface. Increasing the Pd film thickness to 10 nm facilitated better wetting of the Ti substrate by Pd and also increased the number of catalytically active Pd sites for D_2_ adsorption and D-D activation enhancing the D-storage. On the other hand, a further increase in the Pd film thickness to >10 nm yielded a decrease in the D-storage. This latter observation can be attributed to the decreasing surface roughness (Figure 1) as well as the decreasing number of crystallographic disorders/defects on thicker Pd films leading to the increasing coordination number of the Pd sites and decreasing catalytic activity.^49^ Furthermore, increasing Pd film thickness can also hinder the efficient diffusion of D atoms to the underlying Ti film due to the increasing transport path length between the Pd surface and the underlying Ti surface.

**Figure 8:**
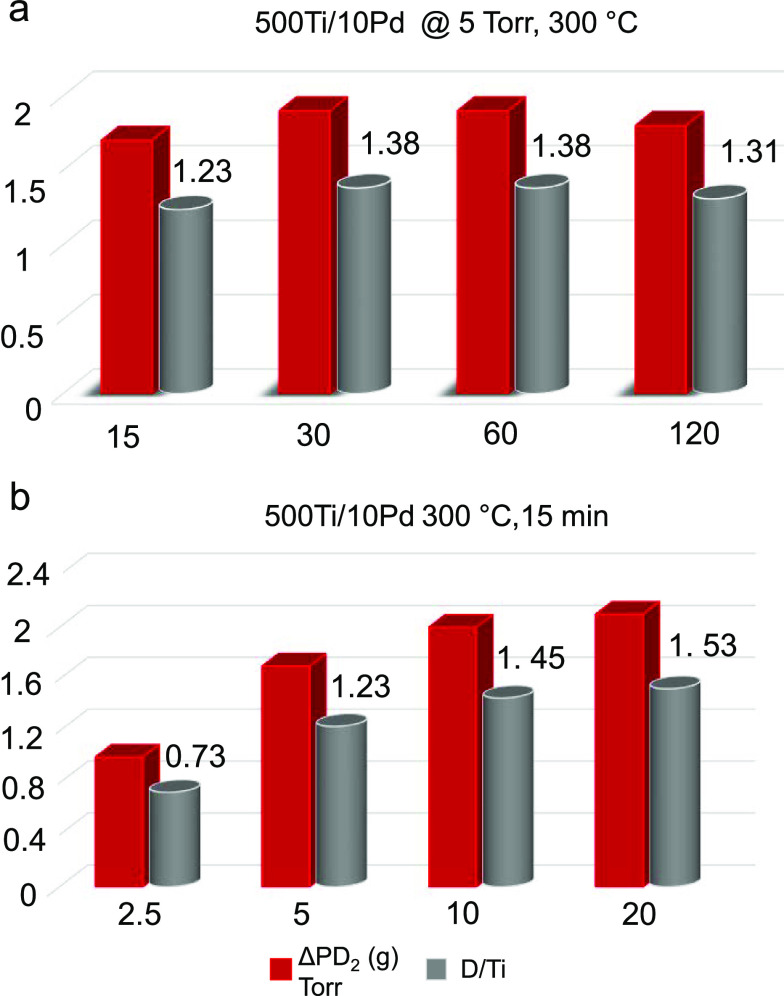
Change in partial pressure of deuterium (Δ*P*_D2(g)_) and D/Ti atom ratio during D_2_(g) storage experiments on 500Ti/10Pd at 300 °C. (a) Influence of D_2_(g) exposure duration (i.e., 15–120 min) at *P*_D2(g)_ = 5 Torr and (b) Influence of *P*_D2(g)_ (i.e., 2.5–20 Torr) at a D_2_(g) exposure duration of 15 min.

As mentioned in Section 2, while the TPD technique was extremely informative for accurate comparison of *relative* amounts of deuterium stored in different thin films as well as in which chemical form deuterium was stored, it failed to provide information on the *absolute* amounts of deuterium stored, which is essential for the estimation of the actual chemical stoichiometry of titanium deuteride (TiD_*x*_) phases that are formed after deuteration. Thus, we prepared larger surface area samples of 2.0 cm × 2.0 cm and measured Δ*P*_D2(g)_ at the beginning and end of the deuteration process to obtain TiD_*x*_ stoichiometry (i.e., D/Ti) as presented in Figure 8. We also used this opportunity to explore whether the deuteration process time can be minimized further by elevating *P*_D2_(g) (Figure 8a,b). It can be seen in Figure 8a that the ultimately optimized sample in the TPD runs (i.e., 500Ti/10Pd, 5 Torr, 300 °C, 2 h) yielded a Δ*P*_D2(g)_ = 1.81 Torr and D/Ti = 1.31. Figure 8a shows that decreasing the deuteration time of the optimized 500Ti/10Pd sample at *P*_D2_(g) = 5 Torr and 300 °C from 2 to 0.25 h decreased the Δ*P*_D2(g)_ and D/Ti. On the other hand, as can be seen in Figure 8b, even for this short deuteration time of 0.25 h, Δ*P*_D2(g)_ and D/Ti values can be increased to 2.1 Torr and 1.53 by increasing the *P*_D2_(g) to 20 Torr, respectively. In light of these findings, it can be argued that in the mass production of tritium-based BV battery systems, tritium storage steps can be significantly accelerated by using *P*_T2_(g) > 5 Torr, as long as the increase in the process cost due to the tritium losses originating from decreasing tritium recovery efficiency at elevated tritium pressures is addressed.

### 3.5 Extending Deuterium Storage Optimization Parameters of Ti/Pd Thin Films to Tritium Storage of Realistic BVB Devices

**Figure 9:**
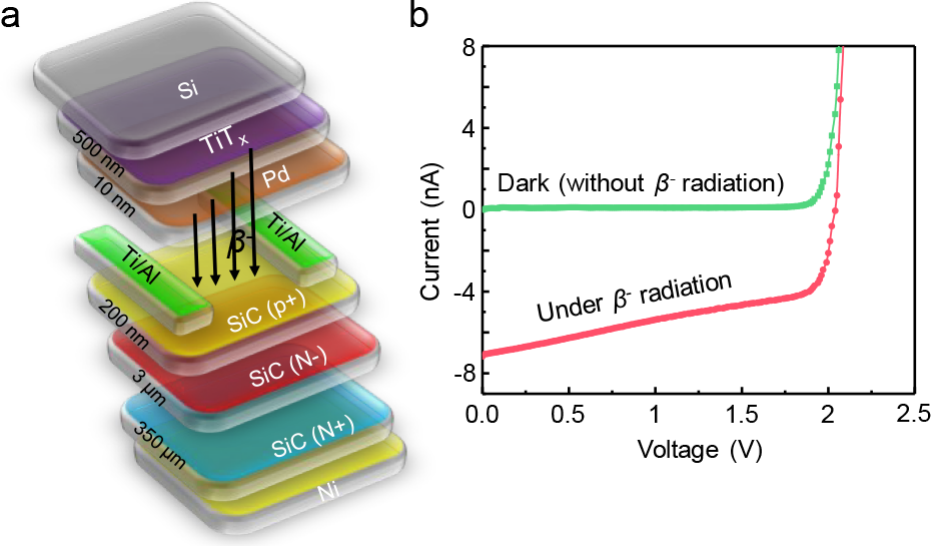
(a) Conceptual description of the BVB device architecture produced after nanofabrication. (b) *I*–*V* curves of the BVB device with and without β*-*particle irradiation source (see text for details).

In order to demonstrate that the currently presented deuterium storage optimization runs presented above are also relevant for the tritium storage of Ti/Pd thin film systems, we exposed a 500Ti/10Pd sample to 5 Torr T_2_(g) at 300 °C for 2 h and incorporated this thin film to a tritium-based BVB device (as shown schematically in Figure 9a). Upon tritium storage, 500Ti/10Pd thin film yielded a promising β*-*particle emission activity of 370 mCi corresponding to a surface area-normalized irradiation activity of 164 mCi/cm^2^.^8,51^ Current–voltage (*I*–*V*) plots obtained during the operation of this tritium-based realistic BVB device is shown in Figure 9b, where the clearly different *I*–*V* responses of the BVB device with and without β*-*particle irradiation are visible. A β*-*generated short circuit current (*I*_SC_) of 7.2 nA and an open circuit potential (*V*_OC_) of 2.04 V can be extracted from the device *I*–*V* response. The contact area is 0.16 cm^2^. The top view optical image of the BVB device is shown in Figure S4. Detailed device characteristics of this BVB module using various Ti/Pd β*-*emitter sources obtained under different titration conditions will be reported in a forthcoming study.

## 4 Conclusions

In this study, we utilized D_2_(g) as a proxy for exploring the tritium storage properties of Pd-coated Ti ultra-thin films at relatively low pressures. We showed that the total amount of deuterium storage, thermal stability, and chemical nature of the stored deuterium species, electronic interactions between D–Pd, and Ti–D can be fine-tuned via careful control of thin film thicknesses and D-storage conditions.

We illustrated that a D/Ti ratio of up to 1.53 could be achieved in the Ti film of the Ti/Pd multimetallic ultra-thin film system, where strong Ti^*x*+^–D^*y*–^ electronic interactions led to the formation of crystallographically well-defined TiD_*x*_ domains with high thermal stability. In stark contrast, D-storage in the Pd thin film component was found to be extremely limited due to the weak interaction between Pd^0^ and D species, where D-containing species resided mostly on the surface of the Pd film rather than bulk Pd, resulting in low thermal stability.

Parametric optimization of the D-storage process over Ti/Pd thin film surface revealed that D-storage typically increased with increasing Ti film thickness, P_D2_, *T*, and *t.* On the other hand, D-storage was observed to vary strongly with the alterations in the Pd film thickness and surface roughness. The optimum Pd film thickness was found to be 10 nm which enabled effective wetting of the underlying Ti film and provided a sufficiently high number of surface defects (roughness) for D immobilization. This particular Pd film thickness also offered a relatively short transport path length for D diffusion from Pd to Ti.

As an ultimate demonstration, we illustrated that the currently used D-storage optimization strategy was also relevant for the tritium-based BVB end-user applications. Along these lines, we showed that tritium T_2_(g) storage on the Pd-promoted Ti ultrathin nano-films incorporated into a realistic BVB device produced promising electron β*-*emission yields of 164 mCi/cm^2^ and reasonable current–voltage (*I*–*V*) curves.

## Supporting Information Available

Details on thin film growth protocols; custom-design D_2_ deposition system; D_2_ deposition protocols; material characterization parameters; additional depth profiling XPS data; and additional TPD profiles (PDF)
