# Supplementary material for: Low-Pressure Deuterium Storage on Palladium-Coated Titanium Nanofilms: A Versatile Model System for Tritium-Based Betavoltaic Battery Applications
Source: ACS Appl Mater Interfaces. 2023 Aug 17;15(34):40459–68. doi: 10.1021/acsami.3c06925 (PMC10472331; doi:10.1021/acsami.3c06925)
Supplement: Supplementary file 1 — am3c06925_si_001.pdf [file am3c06925_si_001.pdf]

SUPPORTING INFORMATION

Low-Pressure Deuterium Storage on Palladium-Coated Titanium Nanofilms: A Versatile Model System for Tritium-based Betavoltaic Battery Applications

Turkan Gamze Ulusoy Ghobadi, Yusuf Kocak, Ahsan Jalal, Yagmur Altinkaynak, Gulsah Celik, Tolga Semiz, Cihan Cakir, Bayram Butun, Ekmel Ozbay*, Ferdi Karadas*, and Emrah Ozensoy*

Turkan Gamze Ulusoy Ghobadi – Nanotechnology Research Center (NANOTAM), Bilkent University, Ankara 06800, Turkey; orcid.org/0000-0002-7669-1587

Yusuf Kocak – Department of Chemistry, Bilkent University, Ankara 06800, Turkey; Nanotechnology Research Center (NANOTAM), Bilkent University, Ankara 06800, Turkey; orcid.org/0000-0003-4511-1321

Ahsan Jalal – Department of Chemistry, Bilkent University, Ankara 06800, Turkey

Yagmur Altinkaynak – Department of Chemistry, Bilkent University, Ankara 06800, Turkey

Gulsah Celik – Nanotechnology Research Center (NANOTAM), Bilkent University, Ankara 06800, Turkey

Tolga Semiz – Nanotechnology Research Center (NANOTAM), Bilkent University, Ankara 06800, Turkey

Cihan Cakir – Nanotechnology Research Center (NANOTAM), Bilkent University, Ankara 06800, Turkey

Bayram Butun – Nanotechnology Research Center (NANOTAM), Bilkent University, Ankara 06800, Turkey

Ekmel Ozbay – Nanotechnology Research Center (NANOTAM), Bilkent University, Ankara 06800; Department of Electrical and Electronics Engineering, Bilkent University, Ankara 06800, Turkey; Department of Physics, Bilkent University, Ankara 06800, Turkey; orcid.org/0000-0003-2953-1828; Email: ozbay@bilkent.edu.tr

Ferdi Karadas – Department of Chemistry, Bilkent University, Ankara 06800, Turkey; National Nanotechnology Research Center (UNAM), Bilkent University, Ankara 06800, Turkey; orcid.org/0000-0001-7171-9889; Email: karadas@fen.bilkent.edu.tr

Emrah Ozensoy – Department of Chemistry, Bilkent University, Ankara 06800, Turkey; National Nanotechnology Research Center (UNAM), Bilkent University, Ankara 06800, Turkey; orcid.org/0000-0003-4352-3824; Email: ozensoy@fen.bilkent.edu.tr

1.1. Materials

Titanium and palladium pellets (purity, 99.9999%) used in the e-beam evaporation process were purchased from Kurt J. Lesker Company, USA. Deuterium (99.9%, 25 L in a 440 mL lecture bottle) was purchased from Cambridge Isotope Laboratories, USA. Si <100> substrates with resistivity of 0-100 ohm.cm was purchased from University Wafer, Inc., USA.

1.2. Ti/Pd Ultra Thin Film Growth

4-inch-diameter Si <100> wafer was ultrasonicated and thoroughly cleaned with high-purity acetone, isopropanol, and distilled water, sequentially; the samples were immersed in 1% HF(aq) for 1 min, and then these cleaned samples were intensively washed with double distilled water. Samples were dried using N_2 gas flow after cleaning. Finally, the clean substrates were mounted into the electron beam evaporator system (Univex 350, Leybold, Germany) for Ti evaporation. Deposition of the Ti or Pd thin-film layers was carried out at an initial chamber pressure of 1 x 10^{-6} mbar with an evaporation rate of 3 Å s^{-1} and 1.2 Å/s for Ti and Pd, respectively. Film thickness was monitored by a quartz crystal microbalance (QCM) installed inside the evaporation chamber, which was adjusted to an accuracy of ±5 nm. The evaporation rate was kept constant within 0.1 Å s^{-1} via a deposition monitor. Pd coating was performed immediately after completing the Ti coating without removal of the sample from the deposition chamber in order to prevent exposure of the sample to any unwanted reactive external gases. Finally, fabricated samples were diced into small pieces of desired sizes.

1.3. Custom-design D_2 Deposition System and D_2 Deposition Protocols

D_2 deposition experiments were carried out using a custom-design system (**Figure S1**) consisting of a deuterium storage reactor (Nanovak R&D Inc., Turkey) equipped with resistive heating and water cooling capabilities, absolute pressure capacitance manometer (MKS, Baratron 626C), ¼ inch VCR bellow valves, an Oerlikon TMP 361 (oil-free) turbo molecular pump, a Varian IDP-7 dry scroll vacuum pump (oil-free, hermetic), an SRS RGA 200 quadrupole mass spectrometer (QMS), D_2 gas cylinder, a UHV hot cathode ionization gauge (BARION BAT35C), Multistage pressure gauge, and a chiller (Nanovak R&D Inc., Turkey) for external cold water circulation.

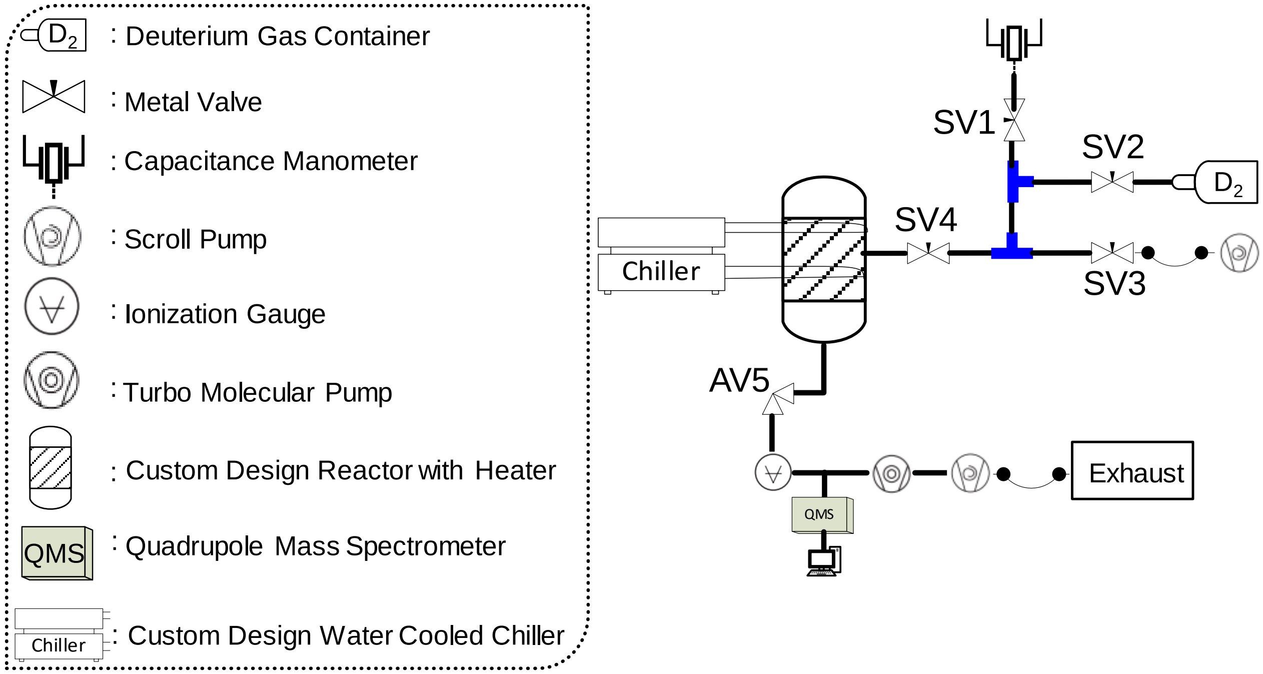

Figure S1. Custom-design D_2 deposition system utilized in the current study.

1.4. Material Characterization

Scanning electron microscopy (SEM) measurements were performed via a field emission gun FEG-SEM (FEI – Quanta 200 FEG). For thin film thickness analyses, a focused ion beam (FIB, FEI Nova600i) system was utilized to prepare cross-sectional samples of the thin films. X-ray photoelectron spectroscopy (XPS) measurements were carried out using a Thermo Scientific K-Alpha XPS spectrometer utilizing Al K_α monochromatic radiation, ($h\upsilon$ = 1486.6 eV, 200 eV pass energy, 1 eV step size) and an electron flood gun for surface charge neutralization. The calibration of the binding energy (B.E.) scale was performed by fixing the adventitious C1s signal at 284.8±0.1 eV. XPS depth profiling studies were carried out by using a rastering Ar^+ ion gun (ion beam energy ca. 1000 eV, raster size 2 mm, sputter rate 0.19 nm/s for Ta_2O_5). The depth profiles of the samples were generated in 100 cycles (duration of each etch cycle was 30 s/cycle). Due to the well-known spectral overlap between $Pd3p_{3/2}$ and O1s XPS features, in the depth profiling studies, oxygen concentration as a function of sputtering time (i.e., film thickness) was determined by exploiting the O2s signal which is less intense than that of O1s signal. In the valence-band XPS (VB-XPS) measurements, a pass energy of 30 eV and a step size of 0.1 eV were used. Grazing-incidence X-ray diffraction (GIXRD) patterns were recorded with a PANalytical X'Pert PRO MRD diffractometer using Cu K_α radiation (45 kV and 40 A). GIXRD data were obtained from 2 cm x 2 cm samples within the 2θ range of 30–90° by the averaging of ten scans, which were performed using 0.08 step size and 18 s counting time. Atomic Force Microscopic characterization was performed using PSIA XE-100 Atomic Force Microscope (AFM) instrument in non-contact mode at ambient temperature with a Si tip (oscillation frequency, 130 - 250 kHz). Root mean square (RMS) roughness and mean roughness (S_a) values were determined on an area of 2 μm × 2 μm per sample and calculated using the Gwyddion analysis software.

2. Additional Data

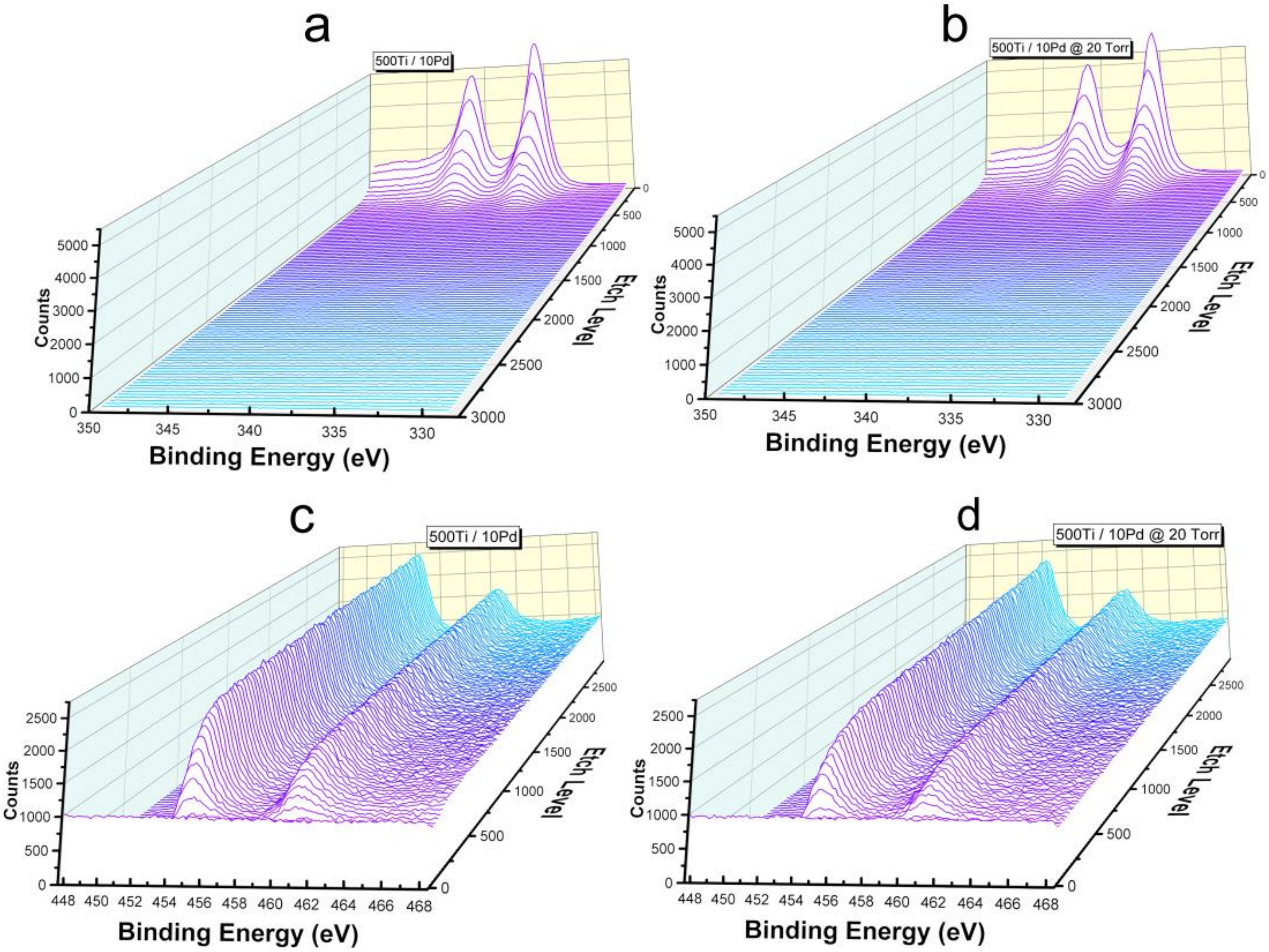

Figure S2. Ti2p (a,b), Pd3d (c,d) depth profiling XPS data as a function of sputtering etch level (0-2800 level) for pristine, and D_2-exposed (20 Torr, 300 °C, 15 min) 500Ti/10Pd thin films, respectively.

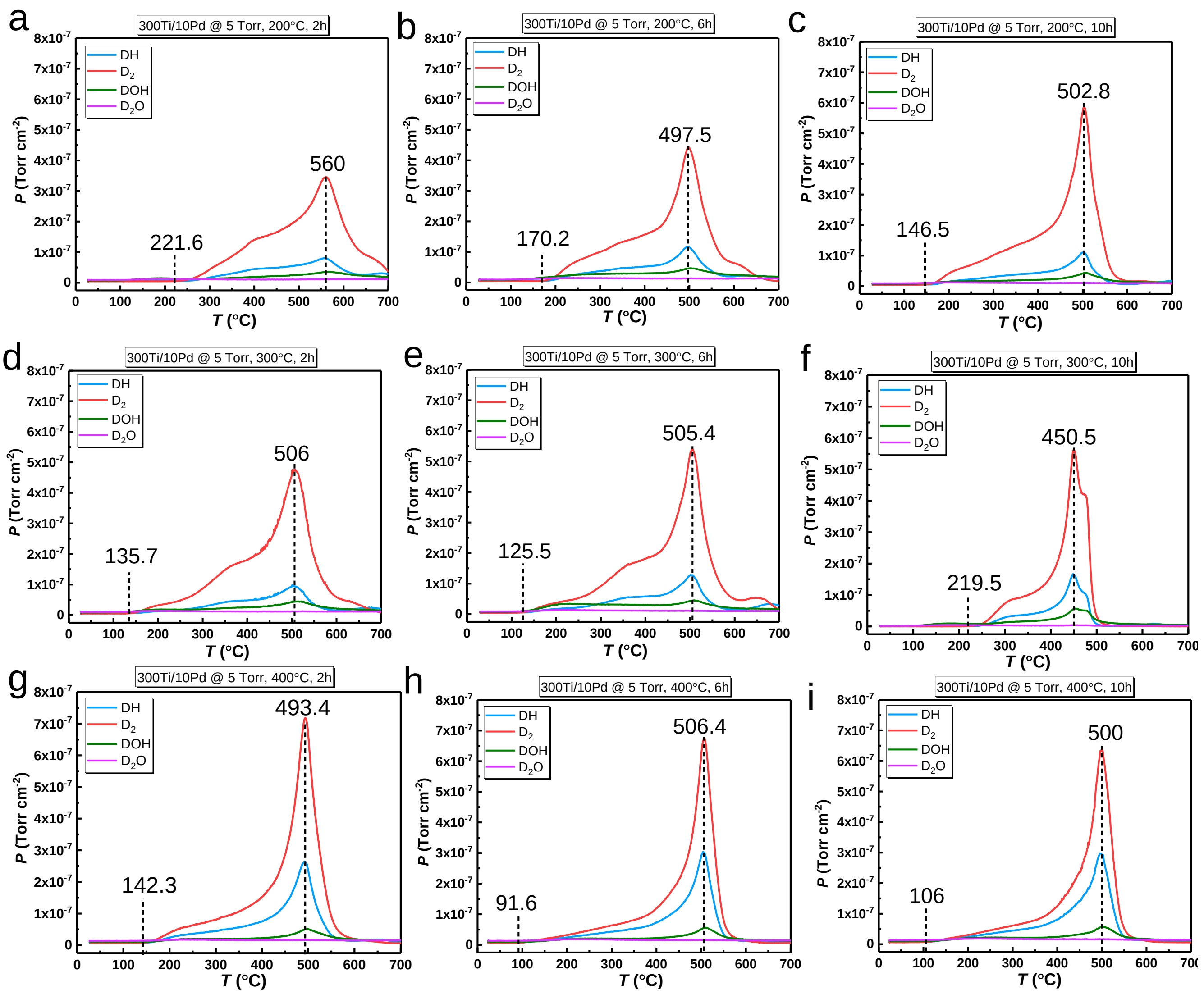

Figure S3. Comparison of TPD Profiles for 300Ti/10Pd where deuterium deposition was carried out at 5 Torr for various durations (2, 6, 10 h), under (a-c) 200 °C, (d-f) 300 °C, and (g-i) 400 °C temperatures.

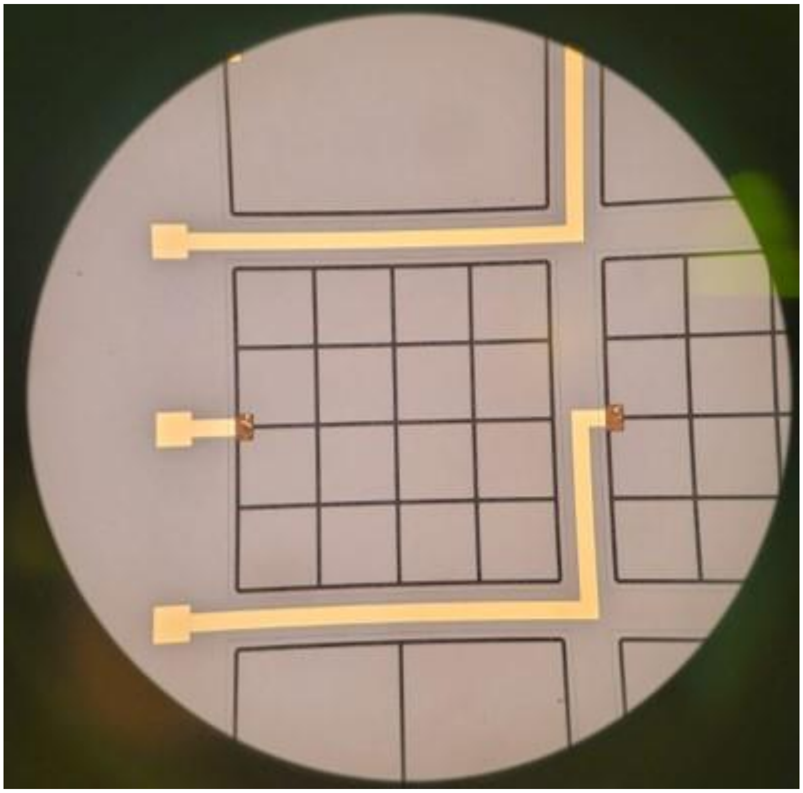

Figure S4. Top view optical image of the BVB device.
